# Results of the epidemiological measurement of endemics, epidemics, and pandemics

**DOI:** 10.1016/j.idm.2026.02.003

**Published:** 2026-02-28

**Authors:** Fabian Standl, Dominik Stelzle, Mirko Trilling, Philipp Jansen, Heribert Stich, Andreas Stang

**Affiliations:** a Institute for Medical Informatics, Biometry and Epidemiology (IMIBE), University Medicine Essen, Essen, Germany; b Technical University of Munich, TUM School of Medicine and Health, TUM Graduate Center of Medicine and Health, Munich, Germany; c Institute of Infectious Diseases and Tropical Medicine, LMU University Hospital, LMU Munich, Munich, Germany; d Institute for the Research on HIV and AIDS-associated Diseases, University Hospital Essen, University of Duisburg-Essen, Essen, Germany; e Institute for Virology, University Hospital Essen, University of Duisburg-Essen, Essen, Germany; f Medical Faculty of the University of Duisburg-Essen, Essen, Germany; g Institute for Medical Information Processing, Biometry and Epidemiology (IBE), Ludwig-Maximilians-Universität München, Munich, Germany; h Pettenkofer School of Public Health, Faculty of Medicine, Ludwig-Maximilians-Universität München, Munich, Germany; i School of Public Health, Department of Epidemiology, Boston University, Boston, USA

**Keywords:** Epidemiology, Virus diseases, SARS-CoV-2, Pandemics, Disease outbreaks

## Abstract

Reliable characterization of infection dynamics is critical for managing endemic, epidemic, and pandemic outbreaks. A persistent challenge in epidemiology is the lack of a unified quantitative framework that allows wave patterns to be compared across outbreak scales and pathogens while accounting for possible ambiguities in wave definitions. This study presents a descriptive epidemiological framework to quantify epidemic wave characteristics using the *Duty Cycle* (DC), defined as the ratio between wave duration and the time to the next peak. Historical time-series data from viral outbreaks were analyzed, and combinatorics was applied to consider all valid interpretations (“perspectives”) of wave boundaries within the same incidence curves, thereby avoiding a perspective-selection bias. We calculated DC values, wave peak postdiction accuracy in days, wave heights relative to initial peaks, and subsequent wave frequencies. Distribution-based analyses show that DC values are generally higher in endemics and epidemics, indicating frequent overlapping waves, whereas pandemics tend to exhibit lower DC and fewer subsequent waves. Wave-peak postdiction accuracy varied across outbreak scales, with distributions reflecting perspective-dependent uncertainty. While subsequent waves of infection in endemics and epidemics are frequently higher than the first wave, pandemics typically peaked in the second wave and showed waning thereafter. The results provide a comparative, structurally grounded description of epidemic wave dynamics across outbreak types, supporting outbreak assessment and planning. Given the heterogeneity of reporting and outbreak dynamics, results should be interpreted as descriptive benchmarks rather than mechanistic postdictions.

## 1 Introduction

Epidemiological measurements of *endemics, epidemics, and pandemics* (outbreak[s]/outbreak scale) and their use have evolved considerably, driven by advancements in data collection and modeling techniques. An in-depth understanding of frequencies and characteristics of *epidemic waves* (wave[s]), including their temporal overlap and recurrence, is crucial for applying effective mitigation strategies. A key challenge in epidemiological measurement is the identification and quantification of infection waves under ambiguous wave-boundary definitions and the postdiction of wave sequences. Using the example of the *SARS-CoV-2* (SARS2)-induced COVID-19 pandemic, there remains uncertainty regarding the precise number and magnitude of infection waves(Anderson et al., 2020; Vespignani et al., 2020). This underscores the necessity for research that enhances predictive capabilities in this regard.

However, current wave-identification approaches often rely on single-peak assumptions or smoothed trend breakpoints, which treat waves as strictly sequential events and implicitly assume a unique wave structure. These approaches can fail to detect situations in which waves partially overlap, e.g., due to regionally asynchronous transmission, behavioral feedback loops, or variant replacement dynamics(Grenfell et al., 2004; Parag, 2021; Parag & Donnelly, 2020; Zhang et al., 2021). Therefore, a unifying and transparent quantitative framework that allows the comparison of wave structures across pathogens and outbreak scales would be very advantageous.

Chowell and colleagues described different pathogen outbreak characteristics based on oscillation properties. This research suggests that the temporal dynamics of epidemic waves can be characterized through specific oscillatory behaviors(Chowell et al., 2019), which may allow for improved forecasting of critical points such as wave onset, peak, and decline. Incorporating these oscillatory characteristics into predictive models could enhance public health preparedness and response efforts(Grassly & Fraser, 2008; Viboud et al., 2016), even in the absence of fully specified mechanistic transmission models. Building upon this foundation, we analyzed an extensive collection of epidemiological time series (=longitudinal) data encompassing outbreaks of various pathogens. To address the ambiguity of wave boundaries, we employ combinatorics to incorporate all equally valid “perspectives” of wave delineation within each time series, thereby avoiding perspective-selection bias and enabling distribution-based summaries of wave characteristics. Our analysis employs the concept of the *Duty Cycle* (DC) to describe oscillation properties in outbreaks of viral diseases. In addition, we investigated the frequency of subsequent infection waves, infection wave heights, and the deviation in days between predicted and observed infection wave peaks.

By providing a comparative, descriptive quantification of wave structures based on distributional summaries rather than a mechanistic model, this study aims to fill the specific knowledge gap of enabling cross-pathogen and cross-scale comparison of epidemic waves. By integrating these measurements, we aim to improve: (I) the understanding of virus infection wave dynamics, (II) accurate epidemic forecasting, and (III) the understanding of the epidemiological nature of virus outbreaks.

## 2 Material & methods

The dataset contains measured incidence curves of endemic, epidemic, and pandemic *perspectives*. We use the term perspectives to describe the various possible interpretations when measuring time series illustrations of virus outbreaks. A perspective is one description of the possible chronological sequence of an incidence curve. An incidence curve consists of one or more epidemic waves. Each wave consists of three characteristic points in time: beginning, peak, and end. A perspective is therefore one specific interpretation of the sequence of these points in time that results from examining an incidence curve. Perspectives arise because there is no clear definition of epidemic waves(Standl, 2023), and it is therefore unclear when exactly an epidemic wave begins or ends(Zhang et al., 2021). Each perspective represents one internally consistent but not unambiguously privileged segmentation of the same incidence curve. Thus, several equally valid wave segmentations can be derived from the same time series.

**Fig. 1:**
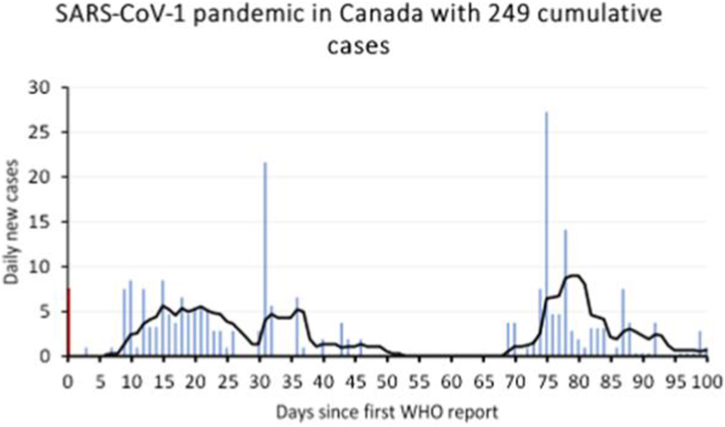
Illustration of multiple valid wave-segmentation perspectives using the SARS-CoV-1 outbreak in Canada (2003). Daily incidence (bars) and 7-day smoothed trend (line) are shown over 100 days since the first WHO report. Depending on how wave onset and offset are defined, the incidence curve can be interpreted as: (I) Two waves, with the first wave peaking around day 15 and the second around day 80; (II) Three waves, where the mid-epidemic rise around day 30 constitutes a distinct wave; or (III) Four waves, if the smaller rise beginning around day 86 is treated as a separate late wave. For each interpretation, multiple start- and end-points are equally credible (e.g., defining wave termination by slope change vs. minimal incidence return), resulting in numerous plausible wave-segmentation perspectives from the same time series. This example demonstrates why selecting a single segmentation introduces subjectivity and supports the use of a combinatorial approach, which incorporates all valid perspectives rather than choosing one. Data source: (Standl et al., 2020); SARS-CoV-1 case surveillance, Canada.

To illustrate how multiple equally valid wave-segmentation perspectives arise in practice, Fig. 1 provides a tangible real-world example; the example is illustrative rather than exhaustive. Depending on the interpretation of where a wave begins and ends, the same incidence curve can be segmented into at least three structurally consistent wave interpretations: (I) A two-wave interpretation: The first wave begins around day 6-8, peaks near day 15, and may be considered to end around day 30, 39, 50, or 53 depending on whether one emphasizes slope changes or returns to baseline. The second wave begins around day 67-73, peaks around day 80, and may end around day 86 or 95 or later. (II) A three-wave interpretation: The first wave begins around day 6-8, peaks at day 15, and ends near day 29. A second wave begins near day 30, peaks near day 36, and ends around day 39, 50, or 53. The third wave then begins around day 67-73, peaks around day 80, and ends on day 86, 95, or later. (III) A four-wave interpretation: As in the three-wave interpretation, but the final rise around day 86-95 is considered a distinct fourth wave, beginning near day 86, peaking near day 88, and ending around day 95 or later. Even within each interpretation, multiple boundary choices are valid. For instance, under the two-wave interpretation alone, there are 4 valid end-points for the first wave x = 2 valid start-points for the second wave x ≥ 2 valid end-points for the second wave, yielding at least 16 valid perspectives. Considering all three interpretation families (two-, three-, and four-wave models) results in dozens of equally plausible wave segmentations derived from the same incidence curve. This example demonstrates: (I) that even under conservative assumptions, dozens of structurally consistent perspectives can arise from a single incidence curve and (II) why selecting a single wave segmentation introduces unavoidable subjective bias. Both points motivate our combinatorial approach, which incorporates all valid perspectives rather than choosing one.

**Fig. 2:**
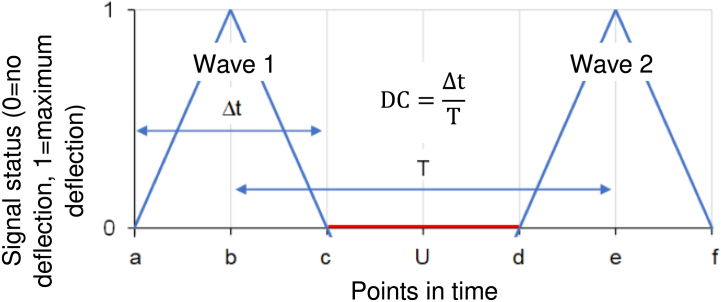
The diagram illustrates the relationship between signal status, points in time, and the timing of two consecutive epidemic waves. The vertical axis shows signal intensity, while the horizontal axis represents events happening in time. Each wave peaks and returns to baseline, with Δt indicating the duration of the elevated signal and T the period. Points a and d show the onset of waves, b and e the peak, and c and f the end of waves. U is the time interval between two waves. The Duty Cycle (DC) reflects the proportion of time the signal remains active within one cycle.

While characterizing outbreaks and their scales may be distorted when reporting the results of random samples, we reduced this bias by aggregating all valid perspectives within country-pairs and treating them as an empirical distribution, rather than selecting a single perspective. For the analysis, we first calculated the DC of waves 1-5 (more than five waves had not been collected in our dataset) per perspective. Waves beyond the fifth were not included because (I) earlier pandemic measurement studies generally analyze only up to three waves, and (II) in our previous preprint work, only the first three to five waves contained sufficient complete data for reliable wave boundary identification(Standl et al., 2020). Thus, the one-to-five-wave horizon represents a practical and historically documented observation window for comparative analysis. The calculation of the DC used is shown in Fig. 2. We use the term postdiction (Nosek et al., 2018) rather than prediction because wave peaks are calculated retrospectively based partially on observed values (Shaman & Karspeck, 2012).

The temporal duration of an epidemic wave is divided by the temporal duration between two points in time with the same phase. In our application, this was the temporal duration between the peaks, i.e., the maximum number of new cases per time unit of an epidemic wave, of two directly consecutive epidemic waves. Numerical values can be assigned to the different points in time (a-f) so that the calculation of DC and different points in time is possible. If, for example, we assign the value 1 to point in time a, the value 2 to point b, and so on, we can calculate this as follows:

$$\text{DC}=\frac{\Delta t}{T}=\frac{\left(c-a\right)}{\left(e-b\right)}=\frac{\left(3-1\right)}{\left(6-2\right)}=\frac{2}{4}=\frac{1}{2}=0.5\text{;}$$

If we want to calculate the peak of the second wave, i.e., time point “e” at a known DC, in our example 0.5, this is possible using the formula:

$$\text{DC}=\frac{\Delta t}{T};T=\frac{\Delta t}{\text{DC}};e-b=\frac{\left(c-a\right)}{\text{DC}};e=\left(\frac{\left(c-a\right)}{\text{DC}}\right)+b=$$

$$\left(\frac{\left(3-1\right)}{0.5}\right)+2=\left(\frac{2}{0.5}\right)+2=4+2=6\text{;}$$

Accordingly, the peak of the second wave is expected at time e = 6. The DC thus quantifies the degree of temporal overlap between successive epidemic waves, with values ≥ 1 indicating that a subsequent wave begins before the previous wave has fully declined.

In the next step, we generated three sub-data sets, one for each outbreak scale. We filtered the sub-datasets and generated lists with unique combinations of the standardized pathogens and the year of the beginning of each outbreak. Pathogen/year selection was based on the availability of sufficiently resolved incidence time series since 1889, the first pandemic with reliable statistical records (Morens & Taubenberger, 2011). To be included in this study, an outbreak needed to allow the identification of at least two valid perspectives of wave segmentation; however, perspectives do not need to contain multiple waves to be part of the dataset. Methodologically, we always count and analyze a maximum of the first five waves from the start of each outbreak, irrespective of whether additional waves occurred later, to ensure comparability across outbreaks.

This dataset is part of an ongoing project that began with pandemic incidence curves and was subsequently expanded to include epidemics and endemics. At the current stage, most included pathogens are communicable viruses causing influenza-like respiratory illnesses, because this is where sufficiently resolved incidence time series and perspective-identifiable curves are most consistently available. We are systematically working through pathogens and published outbreak time series to expand coverage. In the next step of the project, we will incorporate mortality data to complement incidence-based wave characterization. Thus, the present analysis reflects the current state of an evolving empirical taxonomy of wave structures across outbreak scales. Accordingly, the present analysis should be interpreted as an interims assessment of an expanding empirical framework rather than a closed catalog of pathogens.

Since there may be multiple perspectives for an outbreak, each perspective (i.e., each row in the dataset after stratification by pathogen, year, outbreak scale, and country) was treated as one internally consistent realization of epidemic wave structure. In the analytical dataset, each row corresponds to one such perspective for a given country within a pathogen-year-outbreak-scale stratum (country-pair aggregation = pairwise combinatorics). Because multiple perspectives were available per country (each row representing one valid wave-segmentation perspective after stratification by pathogen, start year, outbreak scale, and country), we did not pool all rows across all countries directly. Instead, for each pathogen-year-outbreak-scale group we generated all unique 2-country combinations (country-pairs). For each country-pair, we pooled all perspectives from the two countries and computed pair-level medians for all metrics. We also computed pair-level wave frequency as the percentage of non-missing values at the corresponding wave metrics (filled cells). Wave frequency was operationalized as the percentage of non-missing entries for the corresponding wave-level metrics (i.e., whether a wave peak was observed/recorded in the dataset), summarized at the country-pair level. Finally, we summarized each pathogen-year-outbreak-scale group by reporting the median (Q1; Q3) across the empirical distribution of these country-pair estimates. This approach ensures that no country is counted twice within any combination and reduces dominance of groups with many perspectives from a single country. This pairwise aggregation provides a feasible approximation to full combinatorial enumeration in expectation while remaining computationally feasible for large, multi-country outbreaks. The number of combinations reported in Table 2, Table 3, Table 4 corresponds to the number of country-pairs available for that pathogen-year-outbreak-scale group. In the results, we report the medians (Q1; Q3). We identified the following median DC per outbreak scale and applied these values to calculate the timely postdiction of subsequent wave peaks for each perspective. For the wave pairs from 1 to 2 to 4 to 5, we obtained for endemics: 0.83, 0.75, 0.82, 0.97; epidemics: 0.86, 0.98, 0.86, 0.94; and pandemics: 0.69, 0.67, 0.91, 0.82.

To determine the postdiction accuracy, we used the absolute deviation in days between the observed and the predicted value. Time data collected in weeks or months were converted to a daily resolution. The height of the infection waves was calculated by dividing the heights of the subsequent waves by the height of the respective initial wave. Wave frequency was calculated at the country-pair level as the percentage of non-missing entries for the corresponding wave metrics (filled cells), and summarized as median (Q1; Q3) across country-pairs. We now repeated the iterative approach and calculated the median (Q1; Q3) for: (I) the DC per pathogen and year in which an outbreak occurred, (II) the peak postdiction precision in days, (III) the wave heights in relation to the respective first wave, and (IV) the frequency of subsequent waves in percent.

## 3 Results

**Fig. 3:**
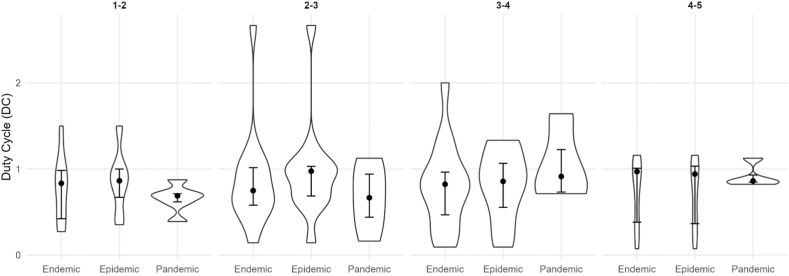
Distribution of Duty Cycle (DC) values across outbreak scales and wave pairs. Violin plots show the distribution of DC estimates for endemic, epidemic, and pandemic outbreaks across consecutive wave pairs (1-2, 2-3, 3-4, and 4-5). Each violin represents the empirical distribution derived from all respective country-pair combinations. Black dots indicate the median DC, and vertical bars denote the interquartile range (Q1-Q3), reflecting variability across equally valid epidemiological perspectives rather than raw incidence variability. Endemic and epidemic outbreaks exhibit higher and broader DC distributions, particularly for early wave pairs, indicating often overlapping waves. In contrast, pandemic outbreaks show consistently lower and more concentrated DC values, especially in later wave pairs, suggesting clearer temporal separation between waves as outbreaks progress.

**Fig. 4:**
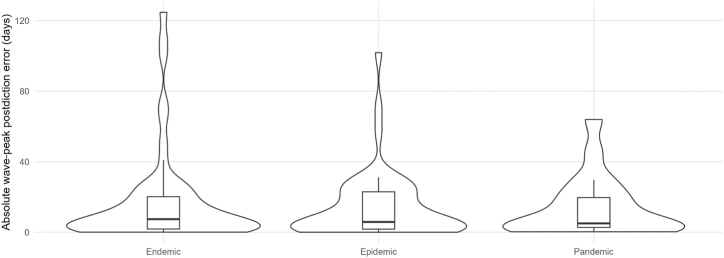
Distribution of absolute wave-peak prediction error in days across outbreak scales. Violin plots illustrate the distribution of absolute deviations (in days) between predicted and observed infection-wave peaks for endemic, epidemic, and pandemic outbreaks. Each distribution is based on the empirical set of country-pair estimates, where each country-pair estimate summarizes all perspectives from the two countries. The embedded boxplots indicate the median prediction error and interquartile range (Q1-Q3), quantifying variability across epidemiological perspectives. Endemic and epidemic outbreaks show broader distributions with pronounced right tails, indicating occasional large prediction errors, whereas pandemics exhibit more concentrated distributions with generally lower median errors, reflecting improved temporal predictability of wave peaks at the pandemic scale despite residual outliers.

**Fig. 5:**
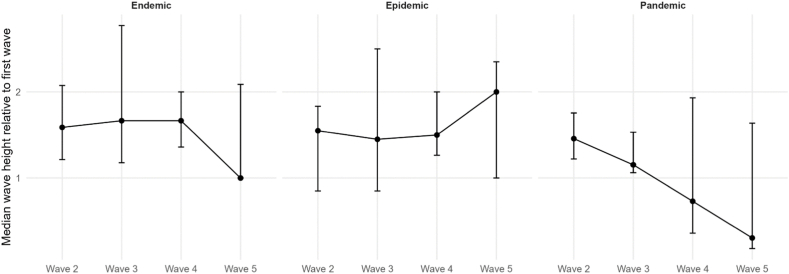
Median relative heights of subsequent infection waves by outbreak scale. Points represent the median height of subsequent infection waves relative to the first wave (wave 1) for endemic, epidemic, and pandemic outbreaks, shown separately by outbreak scale. Error bars denote the interquartile range (Q1-Q3) derived from the distribution of country-pair estimates (medians across all perspectives from each country-pair). Endemic and epidemic outbreaks generally exhibit higher subsequent waves, with median wave heights often exceeding that of the first wave across waves 2-4, whereas pandemic outbreaks show a marked decline in relative wave height from wave 3 onward. This pattern indicates sustained or increasing transmission intensity in en- and epidemics, contrasted with attenuation of later waves in pandemics.

**Fig. 6:**
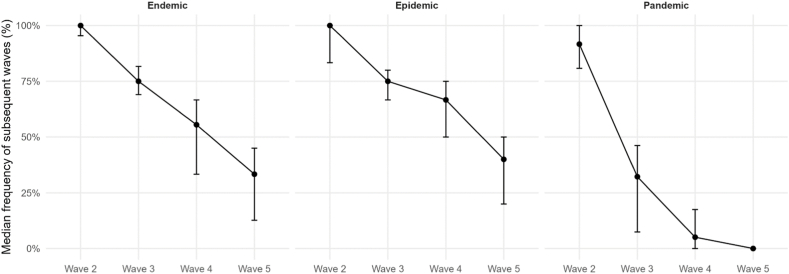
Frequency of subsequent infection waves by outbreak scale. The figure depicts the median frequency of occurrence of subsequent infection waves (waves 2-5) for endemic, epidemic, and pandemic outbreaks in percent. Points represent the median proportion of country-pair estimates in which a given wave was observed (non-missing entries), while error bars indicate the interquartile range (Q1-Q3), reflecting variability across epidemiological perspectives. Endemic and epidemic outbreaks show a gradual decline in the frequency of subsequent waves with increasing wave number, indicating that multiple waves are common but become progressively less frequent. In contrast, pandemic outbreaks exhibit a steep decline in wave frequency after the second wave, with third and later waves occurring substantially less often, highlighting fundamental differences in wave persistence across outbreak scales.

Our analysis comprised 8 different pathogens for endemics, 9 for epidemics, and 4 for pandemics. Our findings reveal distinct epidemiological behaviors across outbreak scales. Table 1 provides an overview, showing that outbreak types have specific wave characteristics. The median DC of endemics and epidemics are a little higher, which corresponds to a higher wave frequency, meaning waves have often not fully declined before the next wave starts. As shown in Fig. 3, these differences are reflected not only in the medians but also in the broader distributions of DC values for endemics and epidemics compared with pandemics. Postdiction accuracy is sufficient for real-world scenarios, as wave peaks can be postdicted with a median absolute deviation of ≤17 days. This is, however, only the case for epidemics, as median postdiction accuracy is ≤ 13 days in endemics and ≤10 days in pandemics. Corresponding to this, median postdiction accuracy can be as precise as 3 days in outbreaks, allowing precise timing, i.a., for public health interventions such as lockdowns or for resource planning regarding medical staff or medical supplies. Fig. 4 illustrates the full distributions of absolute postdiction errors, highlighting substantial variability across outbreak scales and wave phases. We emphasize that postdiction accuracy is reported transparently as absolute deviations in days rather than statistical confidence values to maintain interpretability in applied epidemiological settings. Subsequent waves in endemics and epidemics are usually higher than the first waves, while in pandemics, the fourth and fifth waves are usually smaller than the first waves. The corresponding distributions of relative wave heights are shown in Fig. 5. The frequency of subsequent waves in endemics and epidemics declines comparably to each other, while third waves in pandemics are about half as common as in endemics and epidemics. Fig. 6 visualizes this decline in subsequent-wave frequency across outbreak scales.

**Table 1:** Summary of epidemiological measurements across endemics, epidemics, and pandemics.

Type	Number of pathogens; unique pathogens (sum of country-pairs)	Median Duty Cycles of the respective waves				Absolute values of median peak postdiction precision of the respective wave in days				Median wave heights in relation to first waves				Frequency of subsequent waves in %			
		1-2	2-3	3-4	4-5	2	3	4	5	2	3	4	5	2	3	4	5
Endemic	15; 8 (23)	0.8 (0.4; 1.0)	0.8 (0.6; 1.0)	0.8 (0.5; 1.0)	1.0 (0.4; 1.0)	5 (2; 1)	10 (4; 18)	13 (3; 26)	3 (2; 21)	1.6 (1.2; 2.1)	1.7 (1.2; 2.8)	1.7 (1.4; 2.0)	1.0 (1.0; 2.1)	100 (95; 100)	75 (69; 82)	56 (33; 67)	33 (13; 45)

Epidemic	13; 9 (26)	0.9 (0.7; 1.0)	1.0 (0.7; 1.0)	0.9 (0.6; 1.1)	0.9 (0.4; 1.0)	6 (1; 12)	5 (3; 14)	17 (2; 27)	4 (2; 21)	1.6 (0.9; 1.8)	1.5 (0.9; 2.5)	1.5 (1.3; 2.0)	2.0 (1.0; 2.4)	100 (83; 100)	75 (67; 80)	67 (50; 75)	40 (20; 50)

Pandemic	6; 4 (18,579)	0.7 (0.6; 0.7)	0.7 (0.4; 0.9)	0.9 (0.7; 1.2)	0.9 (0.9; 0.9)	10 (2; 19)	5 (3; 30)	10 (5; 18)	3 (2; 8)	1.5 (1.2; 1.8)	1.2 (1.1; 1.6)	0.7 (0.4; 1.9)	0.3 (0.2; 1.6)	92 (81; 100)	32 (7; 46)	5 (0; 18)	0

This table summarizes key epidemiological measurements across endemic, epidemic, and pandemic waves. Reported are the number of pathogens analyzed, the sum of country-pairs included, median Duty Cycles (DC) for wave pairs, wave peak postdiction accuracy in days, wave heights relative to the first wave, and the frequency of subsequent waves. Numbers, except the number of pathogens and sum of country-pairs, are reported as Median (Q1; Q3). DC values for wave peak postdictions for each wave pair from 1 to 2 to 4 to 5 were for endemics: 0.83, 0.75, 0.82, 0.97; epidemics: 0.86, 0.98, 0.86, 0.94; and pandemics: 0.69, 0.67, 0.91, 0.82. The number of combinations reported corresponds to the number of country-pairs available for that pathogen-year-outbreak-scale group.

**Table 2:** Summary of epidemiological measurements across endemics.

Pathogen	Country-pairs	Median Duty Cycles of the respective waves				Absolute values of median peak postdiction precision of the respective wave in days				Median wave heights in relation to first waves				Frequency of subsequent waves in %			
		1-2	2-3	3-4	4-5	2	3	4	5	2	3	4	5	2	3	4	5
Adenovirus 2020	1	1.0	0.6	0.4	0.4	14	19	27	3	1.0	2.0	5.0	1.0	100	80	60	20

Adenovirus 2009	1	1.0	0.6	0.7	1.0	5	52	11	2	1.8	1.7	1.0	1.0	100	100	33	33

Coronavirus 2020	1	1.5	0.1	0.8	0.1	3	11	0	25	0.3	3.7	0.3	1.0	100	100	75	75

Coronavirus 2009	1	0.3	0.8	0.8	1.0	4	1	0	0	2.6	2.4	2.0	0.4	86	71	29	14

Measles 2015	1	1.5	0.7	0.9	1.0	73	10	6	2	0.9	0.8	1.4	1.0	100	80	60	40

Measles 1968	3	0.8 (0.8; 0.9)	1.0 (0.9; 1.1)	1.3	1.1 (0.9; 1.3)	1	2 (1; 2)	2	1	0.8 (0.5; 1.1)	0.3 (0.3; 0.4)	4.1 (2.8; 5.4)	2.1 (1.6; 2.5)	100	75 (71; 88)	33 (29; 50)	33 (29; 50)

Metapneumovirus 2020	1	0.5	0.6	0.3	0.2	6	8	15	16	1.6	1.5	1.5	1.0	67	67	33	33

Metapneumovirus 2009	1	0.3	0.5	N.A.	N.A.	110	125	N.A.	N.A.	2.8	1.2	N.A.	N.A.	100	50	N.A.	N.A.

Parainfluenza 2020	3	0.9 (0.9; 1.0)	1.0 (0.8; 1.0)	0.6 (0.6; 0.8)	0.7 (0.7; 1.3)	1 (1; 11)	1 (1; 13)	1 (1; 11)	2 (2; 28)	1.9 (1.9; 2.0)	7.5 (6.3; 7.9)	1.7 (1.7; 2.2)	2.2 (1.9; 2.4)	100	71 (69; 77)	71 (69; 77)	71 (69; 77)

Parainfluenza 2009	3	1.0 (0.8; 1.1)	0.8 (0.6; 0.8)	1.3 (0.9; 1.3)	1.2 (1.1; 1.6)	2 (2; 15)	1 (1; 2)	66 (33; 66)	25 (14; 28)	1.6 (1.4; 1.6)	2.8 (2.3; 2.9)	1.7 (1.7; 2.2)	2.0 (1.9; 3.0)	100	83 (83; 92)	50 (42; 63)	33 (25; 54)

Respiratory syncytial virus 2020	1	0.4	1.0	0.2	N.A.	23	41	100	N.A.	5.0	3.0	2.0	N.A.	100	67	67	N.A.
Respiratory syncytial virus 2006	1	0.4	2.7	0.1	0.3	8	8	28	4	2.3	0.5	2.0	2.5	83	50	50	50

Rhinovirus 2020	1	0.8	1.3	1.0	0.9	7	10	15	19	1.6	1.3	1.3	1.0	100	75	75	50

Rhinovirus 2009	3	0.7 (0.5; 0.8)	0.4 (0.3; 0.5)	2.0 (1.7; 2.0)	1.0 (0.8; 1.2)	2 (1; 4)	6 (4; 7)	6 (4; 7)	30 (15; 44)	1.8 (1.8; 1.9)	2.7 (2.6; 2.7)	8.3 (4.7; 8.3)	2.8 (2.3; 3.4)	91 (83; 95)	75 (74; 76)	56 (55; 65)	11 (10; 31)

SARS-CoV-2	1	0.9	1.0	0.9	N.A.	1	17	23	N.A.	1.4	1.2	1.4	N.A.	100	100	67	N.A.

This table summarizes key epidemiological measurements across endemic waves. Reported are the number of pathogens analyzed, the number of country-pairs included, median Duty Cycles (DC) for wave pairs, wave peak postdiction accuracy in days, wave heights relative to the first wave, and the frequency of subsequent waves. Numbers, except the number of pathogens and sum of country-pairs, are reported as Median (Q1; Q3). DC values for wave peak postdictions for each wave pair from 1 to 2 to 4 to 5 were 0.83, 0.75, 0.82, and 0.97. The parasite Malaria was integrated as a sensitivity analysis. N.A. in the table stands for “not applicable” and means that there is no determinable value. This is usually due to the fact that the event that would have generated a value was not observed. The number of combinations reported corresponds to the number of country-pairs available for that pathogen-year-outbreak-scale group.

**Table 3:** Summary of epidemiological measurements across epidemics.

Pathogen	Country-pairs	Median Duty Cycles of the respective waves				Absolute values of median peak postdiction precision of the respective wave in days				Median wave heights in relation to first waves				Frequency of subsequent waves in %			
		1-2	2-3	3-4	4-5	2	3	4	5	2	3	4	5	2	3	4	5
Adenovirus 2020	1	1.0	0.6	0.4	0.4	12	31	27	3	1.0	2.0	5.0	1.0	100	80	60	20

Coronavirus 2020	1	1.5	0.1	0.8	0.1	3	12	2	25	0.3	3.7	0.3	1.0	100	100	75	75

Ebola 2014	10	1.0 (1.0; 1.2)	1.0 (0.7; 1.2)	1.0 (0.7; 1.5)	1.1	2 (2; 3)	1 (1; 3)	1 (0; 2)	0	0.7 (0.6; 0.9)	0.9 (0.4; 1.7)	0.6 (0.6; 1.8)	7.0	82 (75; 87)	32 (22; 45)	20 (16; 29)	0 (0; 6)

Measles 2015	1	1.5	0.7	0.9	1.0	71	5	5	3	0.9	0.8	1.4	1.0	100	80	60	40

Measles 1968	3	0.8 (0.8; 0.9)	1.0 (0.9; 1.1)	1.3	1.1 (0.9; 1.3)	1	2 (1; 2)	2	1	0.8 (0.5; 1.1)	0.3 (0.3; 0.4)	4.1 (2.8; 5.4)	2.1 (1.6; 2.5)	100	75 (71; 88)	33 (29; 50)	33 (29; 50)

Metapneumovirus 2020	1	0.5	0.6	N.A.	0.2	6	8	27	16	1.6	1.5	1.5	1.0	67	67	33	33

Parainfluenza 2020	3	0.9 (0.9; 1.0)	1.0 (0.8; 1.0)	0.6 (0.6; 0.8)	0.7 (0.7; 1.3)	1 (1; 9)	0 (0; 5)	2 (2; 12)	2 (2; 29)	1.9 (1.9; 2.0)	7.5 (6.3; 7.9)	1.7 (1.7; 2.2)	2.2 (1.9; 2.4)	100	71 (69; 77)	71 (69; 77)	71 (69; 77)

Parainfluenza 2009	1	0.7	0.9	1.3	1.2	28	31	57	20	1.6	1.6	1.7	2.0	100	100	75	75

Respiratory syncytial virus 2020	1	0.4	1.0	0.2	N.A.	24	17	102	N.A.	5.0	3.0	2.0	N.A.	100	67	67	0

Respiratory syncytial virus 2006	1	0.4	2.7	0.1	0.3	8	5	29	4	2.3	0.5	2.0	2.5	83	50	50	50

Rhinovirus 2020	1	0.8	1.3	1.0	0.9	7	3	17	21	1.6	1.3	1.3	1.0	100	75	75	50
Rhinovirus 2009	1	1.0	0.7	1.3	1.0	1	3	3	28	1.8	2.5	1.0	2.8	75	75	75	50

SARS-CoV-2	1	0.9	1.0	0.9	N.A.	1	14	26	N.A.	1.4	1.2	1.4	N.A.	100	100	67	0

This table summarizes key epidemiological measurements across epidemic waves. Reported are the number of pathogens analyzed, the number of country-pairs included, median Duty Cycles (DC) for wave pairs, wave peak postdiction accuracy in days, wave heights relative to the first wave, and the frequency of subsequent waves. Numbers, except the number of pathogens and sum of country-pairs, are reported as Median (Q1; Q3). DC values for wave peak postdictions for each wave pair from 1 to 2 to 4 to 5 were 0.86, 0.98, 0.86, and 0.94. The parasite Malaria was integrated as a sensitivity analysis. N.A. in the table stands for “not applicable” and means that there is no determinable value. This is usually due to the fact that the event that would have generated a value was not observed. The number of combinations reported corresponds to the number of country-pairs available for that pathogen-year-outbreak-scale group.

**Table 4:** Summary of epidemiological measurements across pandemics.

Pathogen	Country-pairs	Median Duty Cycles of the respective waves				Absolute values of median peak postdiction precision of the respective wave in days				Median wave heights in relation to first waves				Frequency of subsequent waves in %			
		1-2	2-3	3-4	4-5	2	3	4	5	2	3	4	5	2	3	4	5
A(H1N1) Swine Flu	741	0.7 (0.6; 0.9)	0.7 (0.5; 0.8)	0.7 (0.6; 1.0)	1.1 (0.5; 1.2)	3 (2; 6)	3 (2; 7)	2 (1; 9)	4 (1; 34)	1.4 (0.7; 2.1)	1.5 (0.6; 2.5)	1.0 (0.4; 2.9)	0.4 (0.3; 1.0)	83 (73; 94)	35 (21; 57)	10 (0; 24)	0 (0; 5)

A(H1N1) 2nd Russian Flu	1	0.4	N.A.	N.A.	N.A.	20	N.A.	N.A.	N.A.	0.6	N.A.	N.A.	N.A.	100	N.A.	N.A.	N.A.

A(H1N1) Spanish Flu	55	0.7 (0.5; 0.8)	1.1 (0.9; 1.4)	1.1 (0.7; 1.2)	0.9 (0.9; 0.9)	2	3	14 (13; 18)	3 (3; 5)	2.0 (1.6; 2.3)	0.6 (0.3; 1.0)	0.4 (0.3; 0.5)	0.2 (0.2; 0.3)	80 (57; 88)	30 (20; 46)	0 (0; 13)	0 (0; 6)

A(H2N2) Asian Flu	6	0.9 (0.8; 1.0)	0.4 (0.3; 0.5)	1.6 (0.9; 2.1)	0.9	1 (1; 2)	5 (4; 8)	6 (2; 9)	0	1.5 (0.5; 3.7)	1.2 (0.9; 1.9)	0.2 (0.1; 0.2)	0.0	100	86 (71; 100)	29 (26; 32)	7 (0; 14)

SARS-CoV-2	17,766	0.6 (0.4; 0.9)	0.9 (0.6; 1.2)	0.7 (0.5; 1.0)	0.8 (0.6; 1.0)	64 (39; 108)	45 (25; 70)	29 (13; 51)	20 (6; 36)	1.8 (1.0; 5.0)	3.6 (1.4; 11.3)	4.6 (1.5; 16.1)	5.4 (1.5; 13.3)	100 (80; 100)	50 (33; 75)	20 (0; 50)	0

SARS-CoV-1	10	0.7 (0.5; 0.9)	0.2	N.A.	N.A.	17 (16; 17)	30	N.A.	N.A.	1.2 (0.8; 2.0)	1.1	N.A.	N.A.	50 (30; 69)	0 (0; 22)	N.A.	N.A.

This table summarizes key epidemiological measurements across pandemic waves. Reported are the number of pathogens analyzed, the number of country-pairs included, median Duty Cycles (DC) for wave pairs, wave peak postdiction accuracy in days, wave heights relative to the first wave, and the frequency of subsequent waves. Numbers, except the number of pathogens and sum of country-pairs, are reported as Median (Q1; Q3). DC values for wave peak postdictions for each wave pair from 1 to 2 to 4 to 5 were 0.69, 0.67, 0.91, and 0.82. N.A. in the table stands for “not applicable” and means that there is no determinable value. This is usually due to the fact that the event that would have generated a value was not observed. The number of combinations reported corresponds to the number of country-pairs available for that pathogen-year-outbreak-scale group.

Table 2 details endemic diseases, showing that about 38% of median DC are 1.0 and higher. This indicates that -across a substantial fraction of perspectives-a new wave begins before the previous wave has fully declined. These values underscore a high infectiousness of a virus, as observed, e.g., for the endemic outbreaks of Parainfluenza in 2009 and Measles in 1968, with each having 3 out of 4 median DC ≥ 1.0. Median peak postdiction accuracy was very poor in Metapneumovirus 2009, with 110 and 125 days of median peak postdiction precision. Coronavirus 2009 and Parainfluenza 2020 had a very high accuracy with a median deviation of 1 day and less in 6 of 8 postdictions. The median wave height in relation to first waves was smaller than 1.0 in 14%, equal to 1.0 in 14%, and above 1.0 in 68%, indicating that subsequent waves are usually higher than first waves in endemics. Subsequent wave frequency declines about 25% from wave to wave, starting at a 100% frequency of second waves.

With 46% of DC being at 1.0 or higher, epidemic wave dynamics, presented in Table 3, demonstrate a little greater wave frequency than endemics. At least three median DC of 1.0 and above were observed in Ebola 2014, Measles 1968; Rhinovirus 2009. Median peak postdiction precision was very poor for the second wave in Measles 2015 with 71 days and in Respiratory Syncytial Virus 2020 for the fourth wave with 102 days. Very high precision was observed in Ebola 2014 with three median postdiction deviations of 1 day and less. In Measles 1968, two median postdiction deviations of 1 day were observed. Median wave heights in relation to first waves are often higher than in endemics and were higher than 1.0 in 66% of the measurements, lower in 20%, and at 1.0 in 14%. Subsequent wave frequency declines comparable to endemics about 25% from wave to wave, starting at a 100% frequency of second waves (Bjornstad et al., 2002; Koelle et al., 2022).

Pandemics, summarized in Table 4, exhibited substantial differences from endemics and epidemics across many characteristics. Median DC are usually smaller than 1.0, and only 21% were higher. Median peak postdiction precision was generally good, especially for the influenza pandemics. The comparatively poorer precision observed for SARS2 (median 20-64 days) indicates greater variability in wave timing across perspectives during early pandemic phases. Median wave height was higher than 1.0 in 58% of the measurements and smaller in 37%. In 3 out of 5 relevant measurements, second waves were the highest-peaking waves with regard to the respective median. Only SARS2 had continuously increasing median wave heights. The decline in pandemic wave frequency is different from endemics and epidemics, as second waves are very common, but third waves only occur in about 1/3 of the measurements(Koelle et al., 2022; Morens & Taubenberger, 2011).

## 4 Discussion

Our findings demonstrate distinct epidemiological behaviors across endemics, epidemics, and pandemics. All viruses and outbreak scales exhibit specific wave patterns with gradual declines in subsequent wave occurrence. In pandemics, the decline of subsequent wave occurrence is substantially faster than in endemics and epidemics. This accelerated decline in subsequent-wave frequency is summarized in Fig. 6. Endemics and epidemics exhibit relatively stable further wave characteristics with higher DC, leading to more frequent waves that often overlap. These distributional differences are visualized in Fig. 3. Higher DC values indicate the degree of overlapping waves; incorporating multiple valid segmentations ensures this overlap is not artificially removed by selecting a single wave definition. Sudden changes in wave height may reflect increased transmission intensity, potentially associated with rapid infection spread in partially or fully immunologically naive populations. Epidemics, such as Ebola 2014, Measles 1968; Rhinovirus 2009, show the highest frequency/intensity of subsequent waves, with 46% of DC at or above 1.0. In pandemics, median DC are usually below 1.0, with only 16% exceeding this threshold. Postdiction accuracy is generally better in pandemics, with the Asian Flu achieving a median precision of deviation from postdiction to observation of as low as 0 days, while endemic Metapneumovirus of 2009 shows very poor postdiction accuracy with a median postdiction accuracy for the third wave of 125 days. The corresponding distributions of postdiction error are shown in Fig. 4. Subsequent waves are often higher than the first wave in endemics and epidemics; pandemics tend to peak in the second wave, with SARS2 being an exception due to increasing wave heights over time. Fig. 5 summarizes the relative heights of subsequent waves across outbreak scales. The lower postdiction precision observed for early SARS2 waves likely reflects substantial heterogeneity in testing, reporting, and public health responses during the early pandemic phase(Pullano et al., 2021).

Comparing our results to previous studies, we observe similarities and differences. Chowell et al. concluded, based on their modeling approach, that pathogens exhibit distinct oscillation properties (Chowell et al., 2019) that we also observed in real-world data. However, our findings extend these models by incorporating a substantially broader range of pathogens and by systematically quantifying wave characteristics based on real-world observations. In contrast to model-based approaches, our framework emphasizes distributional summaries derived from empirical incidence data. Vespignani et al. argued that “measuring only the large outbreaks leads to immeasurably large bias.”(Vespignani et al., 2020) This known source of bias was addressed by us using relations and thus making outbreaks of different magnitudes comparable and unveiling the epidemiological characteristics displayed here. Research by Bjørnstad et al. shows that short-term predictability of the dynamics increased with population size(Bjornstad et al., 2002). We did not observe this limitation in our postdiction analyses using the DC framework, likely because the at least pairwise, perspective-based aggregation reduces sensitivity to population size. Davies et al. explored heterogeneity in SARS2 transmission primarily due to age(Davies et al., 2020), which may account for some of the fluctuations seen in our dataset. Undoubtedly, it would make sense to add age and sex information to our existing dataset, as there may be age- or sex-specific characteristics to be described. Grassly and Fraser stated the importance of delivering “parameters of models that describe a disease's natural history and transmission.”(Grassly & Fraser, 2008) Koelle et al. emphasized the limitations of infection epidemiology in SARS2 to be that assumptions heavily relied upon experiences with SARS-CoV-1, MERS-CoV, and, at that time, limited data of SARS2(Koelle et al., 2022). Neher et al. stated the uncertainty of the input parameters for modeling at the beginning of the SARS2 pandemic. There was, for example uncertainty with the amount of epidemic waves, the timing of peak transmissibility, and the range of values for input(Neher et al., 2020). We addressed the issues raised by Grassly and Fraser, Koelle and colleagues, and Neher et al. and provide median values with the corresponding IQR, which narrows down the uncertainty of real-world measurements for many different pathogens and outbreaks. Interquartile ranges reflect variability across country-pair estimates and across perspective ambiguity within those pairs, rather than sampling uncertainty from probabilistic sampling. With our extensive measurements, epidemiologists can now use both pathogen- and outbreak-specific incidence parameters. Baker and colleagues, Kissler et al., and Koelle and coauthors expected non-pharmaceutical interventions during the SARS2 pandemic to result in fewer infections, particularly during periods of stringent infection control measures such as social distancing. Furthermore, the authors expected to observe more infections to occur during the course of the pandemic when restrictions were lifted as a consequence of increasing immunity against SARS2 in the population, which prevents life-threatening diseases but not necessarily infections(Baker et al., 2020; Kissler et al., 2020; Koelle et al., 2022). Our SARS2 results show increasing median incidence wave heights across successive waves, consistent with expectations under changing immunity landscapes and non-pharmaceutical intervention dynamics. This interpretation is further supported by increasing stability in wave-peak timing in later pandemic phases, as reflected in narrower postdiction error distributions. However, we explicitly acknowledge that the increasing wave heights in SARS2 may partly reflect observational bias due to testing availability, and that this could be improved in future work by shifting the analysis from incidence to mortality data where feasible.

Our study has several strengths. The use of combinatorial pairwise aggregation substantially reduces selection bias that would arise from random sampling or from privileging a single country or perspective. Additionally, our dataset includes a wide range of pathogens, enhancing the generalizability of our results. However, some limitations should be acknowledged. First, while we analyze multiple perspectives of epidemic waves, the lack of a universally accepted definition of wave onset and offset leads to some degree of subjectivity in the real-world application of our DC framework. The integration of all valid perspectives intentionally increases interquartile ranges, reflecting epistemic uncertainty rather than measurement error. Second, due to differences in surveillance quality across pathogens and historical periods, reporting heterogeneity may influence the measured wave heights and spacing; this limitation is inherent in all historical outbreak data and is therefore addressed here by focusing on relative rather than absolute values.

## 5 Conclusions

Our research provides a comprehensive framework for understanding viral incidence wave dynamics across different pathogens and outbreaks, reinforcing the need for pathogen-specific epidemiological models and research. The implications of our findings are relevant for both epidemiological modeling and public health policy. Understanding the distinct wave behaviors of different pathogens and outbreaks can help inform targeted intervention strategies. For instance, our analysis highlights distinct wave-height dynamics in SARS2 compared with other pandemic pathogens. The higher frequency of subsequent waves in endemics and epidemics underscores the need for continuous surveillance and adaptive response strategies, particularly in settings with immunologically (at least partially) naive populations. Wave peak postdiction with acceptable precision, particularly in pandemics, can enhance predictions for the timing of interventions like lockdowns and medical resource planning. Multiple-wave outbreaks are not an exception but the rule, and their wave characteristics can be quantitatively expressed together with an explicit measure of uncertainty, represented here by interquartile ranges derived from multiple valid epidemiological perspectives.

It is important to note that the DC is a comparative structural measure and does not directly estimate transmission parameters such as the basic reproduction number (R_0_) or infection and recovery rates. Instead, DC reflects the proportion of time during which incidence remains elevated within a peak-to-peak cycle and therefore provides information complementary to mechanistic models rather than replacing them.

Because the dataset includes historical outbreaks observed under heterogeneous surveillance systems, variation in reported incidence levels and wave heights is unavoidable. This constraint is addressed by focusing on relative rather than absolute measures and by explicitly incorporating all valid wave-segmentation perspectives instead of privileging a single interpretation.

Future research may focus on: (I) identifying further quantitative sources of historic outbreaks that can be integrated into our existing data set and (II) checking if the DC framework can reliably forecast time points like the beginning and end of epidemic waves. Additionally, subsequent work should evaluate the DC framework against alternative analytical approaches (e.g., sub-epidemic decomposition, wavelet spectral analysis, or mechanistic compartmental models) to further clarify performance strengths and use cases. By addressing these gaps and providing a systematic, distribution-based analysis of epidemic wave progression, this work contributes to improved outbreak assessment, preparedness, and evidence-informed decision-making across outbreak scales.

## CRediT authorship contribution statement

**Fabian Standl:** Writing – review & editing, Writing – original draft, Visualization, Software, Resources, Project administration, Methodology, Investigation, Formal analysis, Data curation, Conceptualization. **Dominik Stelzle:** Writing – review & editing, Writing – original draft, Visualization, Validation, Methodology, Investigation, Formal analysis. **Mirko Trilling:** Writing – review & editing, Writing – original draft, Visualization, Validation, Supervision, Methodology, Investigation. **Philipp Jansen:** Writing – review & editing, Writing – original draft, Investigation, Formal analysis. **Heribert Stich:** Writing – review & editing, Writing – original draft, Validation, Supervision, Resources, Investigation, Formal analysis. **Andreas Stang:** Writing – review & editing, Writing – original draft, Visualization, Supervision, Resources, Project administration, Methodology, Investigation, Conceptualization.

## Data statement

The data is available on request from the corresponding author.

## Declaration of competing interest

The authors declare that they have no known competing financial interests or personal relationships that could have appeared to influence the work reported in this paper.
